# Clinical Utility of Measuring Inspiratory Neural Drive During Cardiopulmonary Exercise Testing (CPET)

**DOI:** 10.3389/fmed.2020.00483

**Published:** 2020-09-18

**Authors:** Nicolle J. Domnik, Emil S. Walsted, Daniel Langer

**Affiliations:** ^1^Department of Medicine, Queen's University, Kingston, ON, Canada; ^2^Respiratory Research Unit, Bispebjerg University Hospital, Copenhagen, Denmark; ^3^Research Group for Rehabilitation in Internal Disorders, Respiratory Rehabilitation and Respiratory Division, Department of Rehabilitation Sciences, University Hospital Leuven, KU Leuven, Leuven, Belgium

**Keywords:** inspiratory neural drive, CPET cardiopulmonary exercise testing, diaphragmatic electromyogram EMGdi, respiratory muscles, respiratory disease (RD), chronic obstructive pulmonary disease, diaphragm

## Abstract

Cardiopulmonary exercise testing (CPET) has traditionally included ventilatory and metabolic measurements alongside electrocardiographic characterization; however, research increasingly acknowledges the utility of also measuring inspiratory neural drive (IND) through its surrogate measure of diaphragmatic electromyography (EMGdi). While true IND also encompasses the activation of non-diaphragmatic respiratory muscles, the current review focuses on diaphragmatic measurements, providing information about additional inspiratory muscle groups for context where appropriate. Evaluation of IND provides mechanistic insight into the origins of dyspnea and exercise limitation across pathologies; yields valuable information reflecting the integration of diverse mechanical, chemical, locomotor, and metabolic afferent signals; and can help assess the efficacy of therapeutic interventions. Further, IND measurement during the physiologic stress of exercise is uniquely poised to reveal the underpinnings of physiologic limitations masked during resting and unloaded breathing, with important information provided not only at peak exercise, but throughout exercise protocols. As our understanding of IND presentation across varying conditions continues to grow and methods for its measurement become more accessible, the translation of these principles into clinical settings is a logical next step in facilitating appropriate and nuanced management tailored to each individual's unique physiology. This review provides an overview of the current state of understanding of IND measurement during CPET: its origins, known patterns of behavior and links with dyspnea in health and major respiratory diseases, and the possibility of expanding this approach to applications beyond exercise.

## Introduction

Measuring diaphragmatic electromyography (EMGdi) as a surrogate of inspiratory neural drive (IND) has a tradition extending over 100 years. Its ability to reveal the mechanistic underpinnings of exercise limitation and dyspnea during cardiopulmonary exercise testing (CPET) has popularized its use in research; however, IND is rarely measured in non-research clinical settings. With aims of familiarizing a broad audience with the fundamental principles of IND measurement and its presentation in health and respiratory disease, this review outlines the valuable insights provided by IND measurement during the physiologic stressor of exercise, what these reveal beyond standard testing approaches, and emerging areas of interest in applying IND in diverse research settings. It also reflects on current barriers to the clinical adoption of IND assessment and how these might be overcome.

## Fundamentals of Ind Measurement

### Muscles of Inspiration

The inspiratory muscles fall into two categories: primary (i.e., diaphragm, external intercostal, scalene, and parasternal internal intercostal muscles) and accessory (e.g., sternocleidomastoid, pectoralis minor, etc.) (1, 2). The diaphragm is the foremost driver of inspiration at rest and during exercise, accounting for ~2/3 of lung volume change (3, 4). The scalene and external intercostal muscles show lesser activation during healthy quiet breathing but play an increasingly important role in loaded, high-volume, or distressed breathing patterns (5, 6), while the parasternal internal intercostal muscles are active during resting eupneic breathing, assisting with upper thoracic expansion as well as stabilizing the thorax to the effects of diaphragmatic movement (7, 8). The accessory muscles contribute to inspiration in conditions with higher ventilatory requirements or where breathing pattern is altered (e.g., more rapid) as a result of impaired respiratory mechanics (1). Diaphragmatic IND is the focus of this review. While not discussed herein, the expiratory muscles (i.e., abdominal muscles and internal intercostals) also play an active role in forced exhalations and in supporting the increased ventilation of exercise (9, 10). This is especially critical in conditions of gas trapping, where expiratory recruitment supports subsequent inspiration through elevation of the diaphragm at end-expiration (11, 12).

It is worth noting that rather than being a singular entity, as implied by the nomenclature, the diaphragm consists of two distinct regions: the costal diaphragm, apposing the ribs, and the crural diaphragm, the electrically active region of which is located medially and forms the esophageal hiatus (13, 14). Whereas, the costal diaphragm is involved in the displacement of both abdominal contents and the ribcage, the crural diaphragm displaces abdominal contents only in its caudal, inspiratory descent (13). Thus, the crural diaphragm has a lesser role in thoracic expansion and force generation than the costal diaphragm.

### History of Neural Drive Measurement

EMG measurement via intramuscular needle electrodes has been used to investigate ventilatory mechanisms since the early 20th century (15–19). These earliest observations in dogs and rabbits demonstrated the direct link between phrenic nerve activity and diaphragmatic activation: namely, that action potentials of the phrenic nerve result in electrical activation of the diaphragm (20). Later work ultimately determined the origin of this phrenic activity to be ventilatory drive from the respiratory medulla (21–23). However, the invasive nature and contamination of intramuscular EMGdi with adjacent intercostal muscle activation and breathing movement artifact limited the uptake of this approach in human populations (24). This spurred the development of less invasive techniques using either surface electrodes to measure costal or parasternal EMGdi (25–28) or nasally inserted esophageal catheters to measure crural EMGdi (29–33). Although appealingly non-invasive and relatively easy to use, surface measurements can underestimate EMG activity (vs. esophageal recordings), be contaminated by the electrical activity of neighboring accessory muscles (34–36), or be vulnerable to position and limb muscle mobilization (37, 38). By contrast, esophageal measurements of crural EMGdi are relatively robust, but more technically demanding and potentially uncomfortable for patients. However, the authors' own experiences using this technology, as well as the documented experiences of others, support esophageal catheters being well-tolerated by most patients when skillfully utilized (39).

Contemporary catheter designs build off of earlier designs that utilized a single electrode pair (40, 41). These were prone to artifactual changes in EMG activity due to the relative movement of the diaphragm during breathing as compared with the fixed catheter electrode. Current designs employ multiple electrode arrays arranged as overlapping pairs, which help with positioning the electrodes across the electrically active region (EAR) of the crural diaphragm via cross-correlation analysis as well as compensate for movement of the EAR relative to the electrode during breathing (42–44). [For a more detailed review of esophageal EMGdi measurement, please refer to Luo et al. (45)].

Recent findings suggest that while crural and costal diaphragmatic activation is similar at rest, costal activation (measured by intramuscular recording) increases disproportionately to crural activation when ventilation increases either voluntarily or involuntarily (46–48). This differs from earlier studies that measured costal activity via surface EMG and found parallel increases in costal and crural activity during increase ventilation; however, this difference in findings may be attributable to the greater contamination of surface costal EMGdi with intercostal and abdominal muscle activity (14–16). Thus, while there is significant methodological appeal in the robustness of relatively non-invasive esophageal measurements, it is worth considering that crural recordings may not fully represent IND to the diaphragm, especially during increased ventilatory demand. Parasternal intercostal surface EMG has also gained recent attention as a potential alternative to esophageal crural measurements of IND (49, 50), with emerging data showing strong congruence in baseline activation and profiles of increasing activation in response to increasing IND between surface parasternal intercostal and esophageal crural measurements (28, 50–53).

### Contemporary Approaches to Measuring Neural Drive

Modern IND assessment increasingly combines multipair esophageal EMGdi with invasive (esophageal/gastric manometry) or non-invasive (please see accompanying review by (54): “Non-invasive evaluation of dynamic respiratory mechanics”) measurement of respiratory mechanics (45, 55). EMGdi now routinely replaces traditional IND estimates during CPET, such as minute ventilation (V_E_), esophageal (Pes), transdiaphragmatic (Pdi), or mouth occlusion pressures (37). While these are influenced by obesity (56) or disease-altered respiratory mechanics (57–61), measuring the initiating contractile signal rather than resulting mechanical response provides a more direct assessment of IND. Measuring centrally originating IND with the resulting mechanical (e.g., Pdi) or ventilatory [V_E_, tidal volume/vital capacity (V_T_/VC), or V_T_/VC_pred_] response of the system additionally enables direct investigation of neuromechanical and neuroventilatory coupling or dissociation, respectively (55, 62). Whereas coupling is used to refer to the efficiency with which the electrical signal is converted into a mechanical or ventilatory response, dissociation refers to EMGdi not translating into a mechanical or ventilatory response as efficiently as in health. While there is some variation in how EMGdi is reported alongside mechanical or ventilatory outcomes between authors, in the present work, these are represented by the commonly employed EMGdi:Pdi and EMGdi:V_T_/VC_pred_, respectively, unless stated otherwise.

Although modern esophageal EMGdi is relatively robust to movement artifact or neighboring muscle activity, two technical notes are warranted. (1) While crural EMGdi necessarily contains electrocardiographic artifact, its regularity and distinct profile allows for ready isolation (visual or computational) from the surrounding respiratory signal (45, 63). (2) Between-individual (or within-individual, when measured during different sessions) differences in electrode: muscle fiber orientation, impedance, muscle blood flow, and distance (or amount of tissue) between electrode and muscle surface necessitate signal standardization (64). As per the values reported in this review, this is typically achieved by presenting EMGdi as a percentage of maximum voluntary activation (EMGdi_%max_) obtained during inspiratory capacity (IC) or sniff maneuvers (65, 66). Such maximum maneuvers show strong between-visit reliability (67); however, it is worth mentioning evidence that EMGdi_%max_ may most appropriately be used to normalize for between-group differences, while normalization to ECG R-wave amplitude or to resting tidal EMGdi may be more reliable when investigating intra-individual, inter-visit differences (68).

## Neural Drive in the Evaluation of the Breathless Patient

### The Spectrum of Normal

In healthy adults, resting tidal EMGdi represents only 7–10% of maximum voluntary activation (39, 69). However, this range belies variations. Resting EMGdi can double to 22%_max_ in obesity, for example, due to increased ventilatory load and effort (Pes) (70). Healthy aging's impact on baseline IND is also an important consideration, especially when assessing individuals with chronic respiratory diseases. This is particularly relevant considering the strong relationship between IND and dyspnea (71, 72), i.e., the “subjective experience of breathing discomfort that consists of qualitatively distinct sensations that vary in intensity” (73). Unlike the V_E_:dyspnea relationship, which is limited when respiratory mechanics are impaired, EMGdi_%max_ robustly correlates with dyspnea in health and across disease severity (50, 74). Dyspnea is thought to reflect awareness of the mismatch that results when increased IND does not or cannot result in an adequate mechanical or ventilatory response (75). While not present during resting tidal breathing in health, the stressor of exercise or pathophysiologic processes of disease typically provoke sensations of dyspnea (76).

Aging induces emphysema-like changes in the lung (increased pulmonary compliance) while decreasing chest wall compliance (77, 78). Aging additionally reduces inspiratory muscle strength, decreases diffusing capacity, decreases the proportion of Type II muscle fibers in the diaphragm, and decreases the number of phrenic motoneurons (79–81). Investigation into whether these changes translate into altered IND found that resting crural EMGdi was 40% greater in individuals > 51 years than those <50 years (39); however, these findings standardized EMGdi to maximum voluntary activation, which may be reduced (e.g., inability to achieve—or motivation to perform—truly maximal maneuvers) (82, 83). Recent work specifically investigating motor unit discharge rate (monopolar needle recording of costal diaphragm) found no changes across age groups at rest, despite neurogenic changes in motor unit potential area and discharge time that may become more relevant at higher ventilation (84). Interestingly, despite known sex differences in pulmonary structure [smaller lungs, narrower airways (85)] and function [increased resistive work of breathing and greater propensity for expiratory flow limitation and exercise-induced hypoxemia (86)], resting EMGdi does not vary between age-matched healthy males and females (87, 88).

### Healthy Responses to Exercise

**Figure 1:**
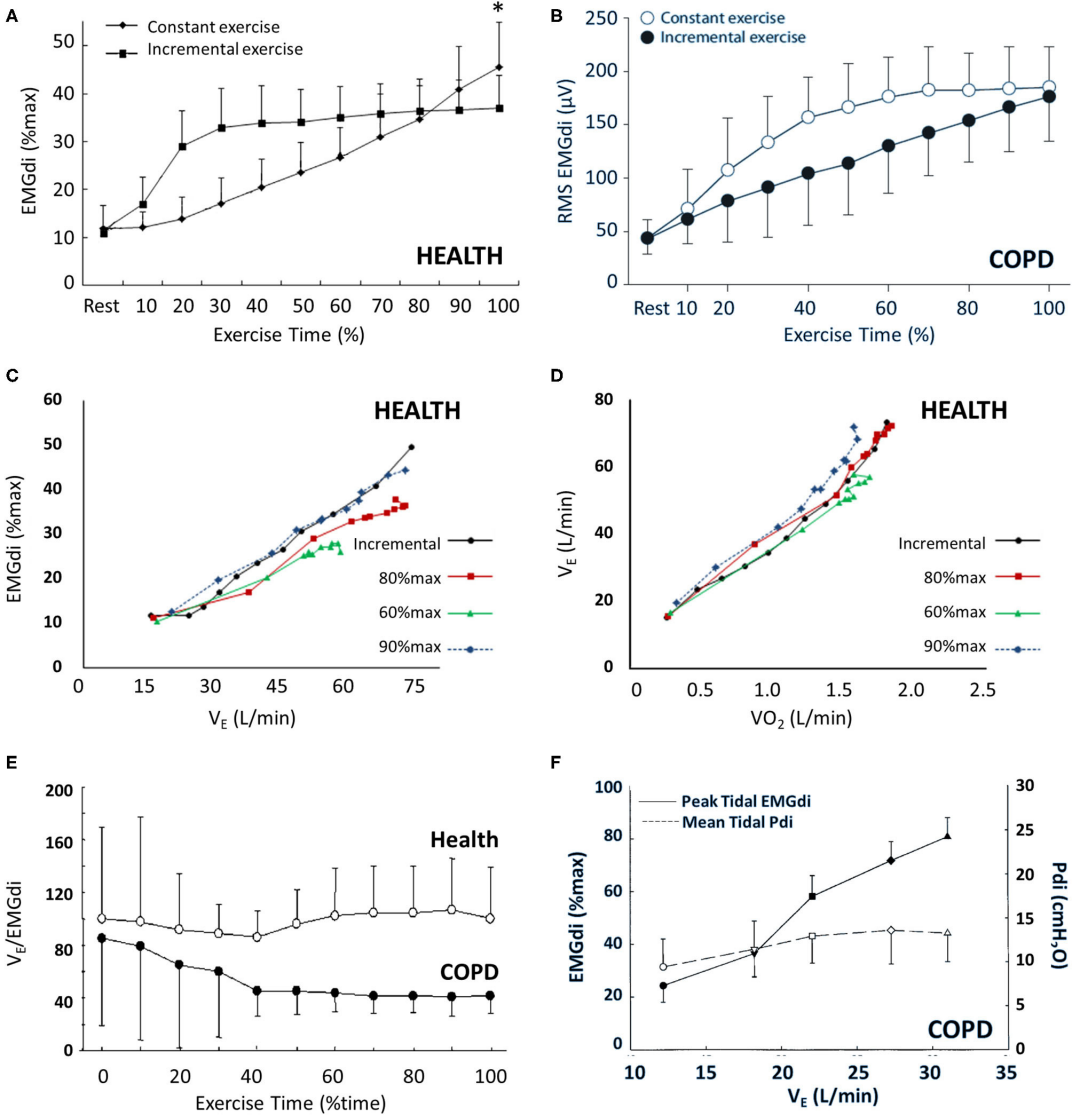
EMGdi behavior during incremental (ICR) and constant work rate (CWR) exercise in health and COPD. Panel **(A)** shows the gradual increase in EMGdi (%max) associated with ICR and the rapid increase in EMGdi (%max) and subsequent plateau associated with CWR exercise in health (*p < 0.05). A similar pattern of behavior is seen in COPD **(B)**. The relationship between EMGdi and V_E_ **(C)** and between V_E_ and VO_2_ **(D)** is maintained regardless of exercise type (ICR vs. CWR) or intensity (CWR at 60, 80, or 90% of maximum work rate). While V_E_/EMGdi is maintained in health during CWR **(E)**, and Pdi/EMGdi is maintained in COPD **(F)**, there is uncoupling of V_E_ and EMGdi in COPD during exercise **(E,F)**. Panels **(A)**, **(C)**, and **(D)** were adapted from (69); panel **(B)** was adapted from (57); panel **(E)** was adapted from (89); and panel **(F)** was adapted from (90). Panels **(A)**, **(C)**, and **(D)** are reprinted from *Resp Physiol Neurobiol*, 189(1), Zhang D, Gong H, Lu G, Guo H, Li R, Zhong N, et al. Respiratory motor output during an inspiratory capacity maneuver is preserved despite submaximal exercise, 87–92, Copyright 2013, with permission from Elsevier. Panel **(B)** is reprinted from *Respiration* 81(4), Luo YM, Li RF, Jolley C, Wu HD, Steier J, Moxham J, et al., Neural respiratory drive in patients with COPD during exercise tests. 294–301, Copyright 2011, with permission from S. Karger AG, Basel. Panel **(E)** is reprinted from *Chest*, 138(6), Qin YY, Steier J, Jolley C, Moxham J, Zhong NS, Luo YM. Efficiency of neural drive during exercise in patients with COPD and healthy subjects, 1309–1315, Copyright 2010, with permission from Elsevier. Panel **(F)** is adapted with permission of the American Thoracic Society. Copyright ^©^ 2020 American Thoracic Society. All rights reserved. Cite: Sinderby C, Spahija J, Beck J, Kaminski D, Yan S, Comtois N, et al. (2001) Diaphragm activation during exercise in chronic obstructive pulmonary disease. *Am J Respir Crit Care Med* 163(7):1637–41. The *American Journal of Respiratory and Critical Care Medicine* is an official journal of the American Thoracic Society. Readers are encouraged to read the entire article for the correct context at https://doi.org/10.1164/ajrccm.163.7.2007033. The authors, editors, and The American Thoracic Society are not responsible for errors or omissions in adaptations.

Two common exercise protocols that are used to study IND are constant work rate (i.e., constant load; CWR), where a constant submaximal output is maintained, and incremental (ICR), where work rate increases in stepwise fashion at predetermined time intervals. The ability of ICR protocols to interrogate the IND profile to the boundaries of maximal exercise capacity offers unique advantages over CWR protocols, including continually increasing IND in concert with continually increasing dyspnea from rest to symptom limitation. This is in contrast with CWR protocols, where IND initially increases before maintaining a submaximal plateau until end exercise (Figure 1A). EMGdi activation during exercise typically plateaus at submaximal values <80%_max_, with some variability reported between studies and populations (55, 69, 89, 90). This begs the question: is this submaximal activation appropriate for the required output or reflective of central inhibition (69, 91, 92)? The maintenance of maximal voluntary IND as achieved through IC maneuver throughout various exercise protocols suggest that the former interpretation of task-appropriate IND is true, rather than neural inhibition (69).

**Figure 2:**
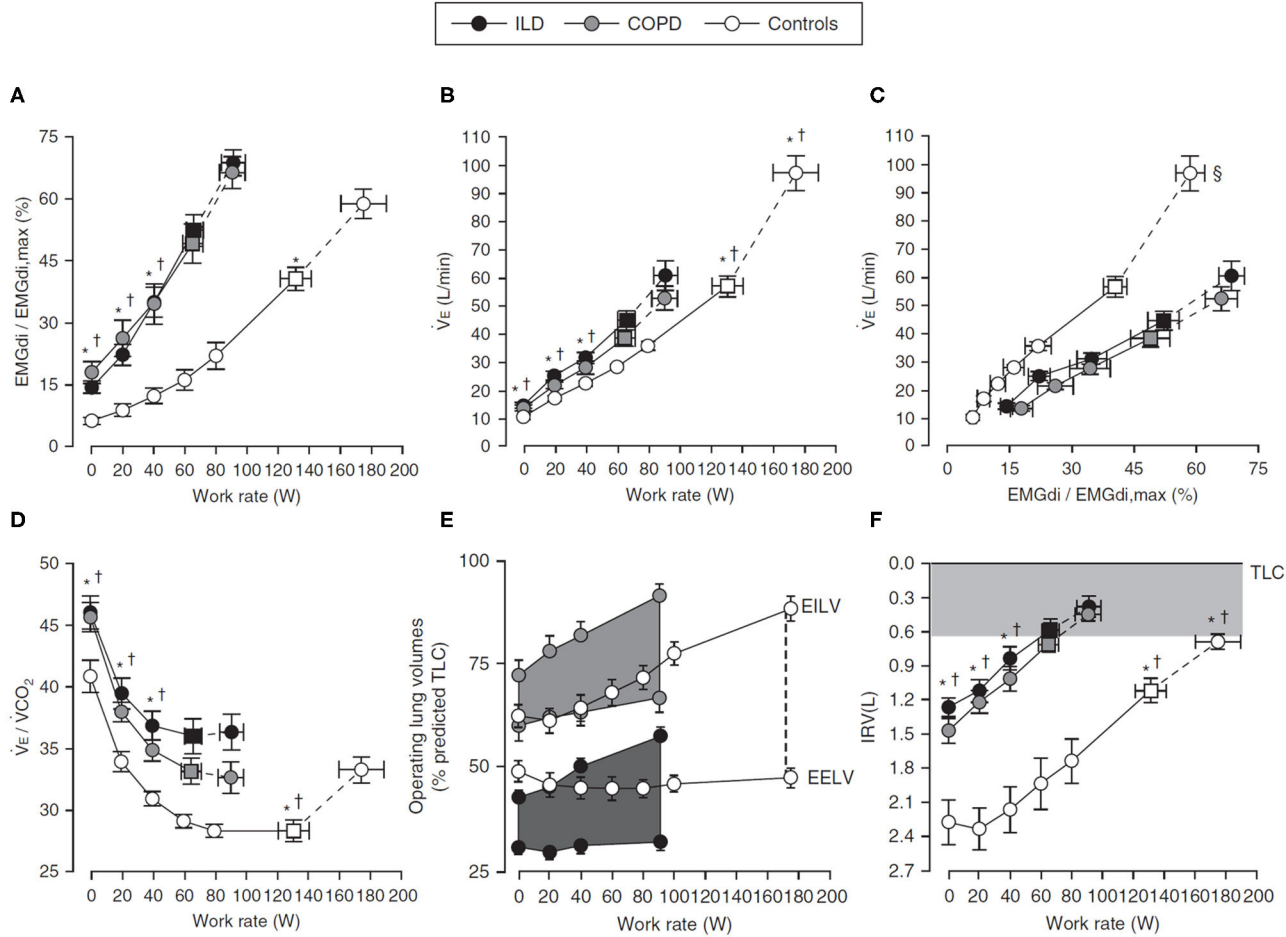
Response to incremental cycle exercise in health (Controls), COPD, and ILD. Values are mean ± SEM, and squares represent V_T_-V_E_ inflection points. *p < 0.05 (ILD vs. Control); †*p* < 0.05 (COPD vs. Control); ‡*p* < 0.05 (COPD vs. ILD); §*p* < 0.05 for for differences in VE/(EMGdi/EMGdi,max) slopes between patient groups and control participants. Panel **(A)** shows IND as EMGdi (%max) increasing throughout ICR, panel **(B)** shows the associated ventilatory response (V_E_), and panel **(C)** shows the coupling of EMGdi with V_E_. Respiratory efficiency is decreased **(D)** (i.e., V_E_/VCO_2_ increased) in respiratory disease relative to Control, in part due to significant ventilatory constraints occurring alongside dynamic hyperinflation [**(E)**, V_T_ expansion during exercise and earlier attainment of inspiratory reserve volume threshold, **(F)**]. Figure adapted from (55). Figure is adapted with permission of the American Thoracic Society. Copyright ^©^ 2020 American Thoracic Society. All rights reserved. Faisal A, Alghamdi BJ, Ciavaglia CE, Elbehairy AF, Webb KA, Ora J, et al. (2016) Common Mechanisms of Dyspnea in Chronic Interstitial and Obstructive Lung Disorders. *Am J Respir Crit Care Med*, 193(3):299–309. The *American Journal of Respiratory and Critical Care Medicine* is an official journal of the American Thoracic Society. Readers are encouraged to read the entire article for the correct context at https://doi.org/10.1164/rccm.201504-0841OC. The authors, editors, and The American Thoracic Society are not responsible for errors or omissions in adaptations.

Ventilation and dyspnea parallel EMGdi during exercise: all three increase with exercise time and intensity [Figures 1C,D; 2A–C; (57, 69)]. Neuroventilatory and neuromechanical relationships (EMGdi relative to V_E_ or Pdi) are maintained throughout exercise in health (69, 89, 90). This is especially relevant in the context of healthy aging, which is accompanied by decreased ventilatory efficiency (i.e., increased V_E_/VCO_2_) and increased ventilatory demand (80). These changes are thought to occur as a result of increased physiologic dead space, i.e., ventilation–perfusion (V/Q) inequalities (93, 94), decreased PaCO_2_ setpoint (95–97), increased anatomic dead space (95), and greater likelihood of terminal airway closure at higher closing volumes (98). Exertional dyspnea also increases alongside loss of static muscle strength in aging, and older females report greater dyspnea than older males for a given absolute V_E_ (99). While you will recall the lack of sex differences in healthy resting EMGdi, exercise protocol type seems to influence the occurrence of sex-specific exercise responses in young adults. Specifically, while EMGdi does not vary between healthy young males and females during CWR protocols performed at the same relative intensity (87), females have higher EMGdi_%max_ and dyspnea for given absolute workloads during ICR exercise (88). This likely reflects the higher ventilation (as a fraction of maximum ventilatory capacity) required to sustain a given absolute work rate in females vs. males (85, 88, 100, 101).

### Neural Drive and Dyspnea Are Elevated in Respiratory Disease

Many respiratory conditions with diverse underlying pathological mechanisms result in elevated resting tidal IND and dyspnea. In chronic obstructive pulmonary disease (COPD), resting IND is increased 2-fold (EMGdi_%max_ >20%) vs. age-matched health (39, 90). Similar magnitudes of increase are seen in interstitial lung disease (ILD) (55, 102) and cystic fibrosis (49). This is linked to pathophysiologic alterations in mechanical and chemical factors (103) and can already appear in very early disease, as detailed in the accompanying review by (104): “Dyspnea and Exercise Limitation in COPD: the value of CPET.”

In obstructive disease, mechanically characterized by increased compliance, gas trapping, hyperinflation, and reduced IC, IND correlates with the severity of airflow limitation (decreased forced expired volume in 1 s; FEV_1_) and degree of hyperinflation (39, 49), due to reduced pressure-generating ability of the diaphragm (105). Mechanical impairment causing increased IND is experimentally supported by acutely increased EMGdi alongside loss of FEV_1_ post-histamine bronchoprovocation challenge in asthmatic children (106). IND is also increased in restrictive diseases like ILD, where decreased compliance and low lung volumes decrease IC. Thus, in both obstructive and restrictive conditions, IND typically increases alongside increasing mechanical impairment (39). Such situations of increased diaphragmatic loading or impairment also increase recruitment of non-diaphragmatic inspiratory muscles (5, 6).

Increased IND can reflect underlying mechanical impairment, but how chemical impairment (e.g., gas exchange abnormalities) might also be reflected is of increasing interest. For example, it has been demonstrated that increased physiologic dead space (i.e., V/Q mismatch), necessitating increased V_E_, reducing ventilatory efficiency, and ultimately resulting in earlier attainment of mechanical constraints, contributes to the increased IND observed in disease (107, 108). This is experimentally supported by increased IND during dead space loading (109) or acute increases in PaCO_2_ in health, with the EMGdi-PCO_2_ relationship suggested as an index of chemosensitivity (68). Data suggest that IND also increases linearly with increasing CO_2_ during rebreathing in COPD (110); however, the impact of chronic hypercapnia on IND and CO_2_ responsiveness in respiratory disease is equivocal. While some groups report blunted CO_2_ responsiveness in hypercapnic COPD (111), others report increased IND in hypercapnic COPD with equivalent mechanical impairment to normocapnic COPD (112). These differences may arise from methodological or group differences (acute CO_2_ exposure vs. chronic hypercapnia; degree of mechanical impairment; analysis of the EMG signal through integration, moving average, or peak) and highlight the need for further studies to clarify the role of chronic hypercapnia, increased physiologic dead space, and diffusion impairment on IND. Increased IND secondary to hypercapnia is likely attributable to a combination of chemosensory inputs, resultant ventilatory changes and the mechanical limitations they precipitate, and afferent signals from mechanically overloaded inspiratory muscles (113). Finally, patients with hypercapnic COPD tend to also experience chronic hypoxia, which may further contribute to IND via chemo-afferent pathways (114, 115) and through diaphragmatic fatigue (116).

### Diaphragmatic Responses to Exercise in Respiratory Disease

Despite different pathophysiologic underpinnings, there is interesting similarity in the diaphragmatic and ventilatory responses to exercise seen in obstructive and restrictive diseases, both of which are exaggerated compared with health [Figures 2A–C; see also (117)]. As in health, baseline IND increases with increasing exercise and metabolic CO_2_ output in respiratory disease (57, 89, 90), either to a plateau in CWR protocols or until end exercise is achieved in ICR protocols [Figure 1B; (57, 89)], but the relative IND is elevated for an absolute work rate vs. health. Further, whereas EMGdi is maintained relative to V_E_ throughout CWR exercise in health, both V_E_ and Pdi gradually decline relative to EMGdi throughout exercise in COPD [Figures 1E,F; (89, 90)], indicative of a declining efficiency of IND during exertion in this population. A similar pattern is seen in the neuromechanical and neuroventilatory dissociation of EMGdi/Pdi and EMGdi_%max_:V_T_/VC_pred_ during ICR, with persistently increasing IND in the face of earlier constraints in increasing Pdi or V_T_.

The higher ventilatory requirements of exercise stress the physiologic tolerances of the respiratory system, exposing underlying impairments. For example, in COPD, baseline CO_2_ retention occurring due to ventilation–perfusion mismatch at rest is further exaggerated during exercise by the inability of the mechanically disadvantaged system to meet the increased metabolic demands of exercise (Figures 2C,D) (118). This, in turn, further increases IND and ventilation. When paired with a rapid, shallow breathing pattern increasing dead space, and underlying expiratory flow limitation leading to dynamic hyperinflation and encroachment of tidal volume on critical inspiratory reserve (Figure 2E) (119, 120), early cessation of exercise and a higher symptom burden for a given work rate ensue (75). In ILD, low diffusing capacity and low pulmonary compliance result in increased ventilatory drive and a rapid, shallow breathing pattern due to limited V_T_ expansion, ultimately also leading to premature termination of exercise and exaggerated dyspnea (55).

The exercise limitations observed in obstructive and restrictive disease are due to an inadequate mechanical response to the higher IND, with the lower IC of both populations limiting V_T_ expansion and causing earlier attainment of the lowest critical inspiratory reserve volume, IRV, and a reliance on increases in breathing frequency to increase V_E_ [Figures 2E,F; (90, 119–121)]. Whether resulting from the hyperinflation-disadvantaged length–tension relationships of the diaphragm (122, 123) and impaired ability to generate inspiratory pressure in situations of increased inspiratory flow (41, 124, 125) in COPD or due to low compliance and low operating lung volumes in ILD, mechanical impairments prevent the efficient translation of drive into ventilatory response. Thus, in both obstructive and restrictive disease, the slope of the relationship between EMGdi_%max_ and work rate is increased relative to health, as is the slope of the dyspnea: work rate relationship (55).

### Using Exercise to Reveal Impairments Hidden at Rest

The utility of CPET as an adjunct to resting pulmonary function testing is further highlighted by respiratory conditions with normal or relatively preserved resting IND, such as exercise-induced laryngeal obstruction (EILO), which presents primarily in young individuals during high-intensity exercise (126). Here, normal resting IND becomes progressively augmented relative to health at increasing work rates, reflecting increasing inspiratory resistive work of breathing, with a significantly elevated IND approaching end exercise (127). Thus, in contrast to the possible beneficial effects of exercise-associated laryngeal closure associated with obstructive pulmonary conditions (128), the laryngeal closure observed in EILO causes both mechanical impairment and increased IND (127). Interestingly, individuals with EILO and those with obstructive pulmonary disease report “unsatisfied inspiration” at high work rates, a convergence of symptoms despite markedly different underlying pathophysiological mechanism contributing to each group's increased IND (117, 127).

IND measurement and CPET are particularly valuable in smokers at risk of COPD and individuals with mild COPD. Despite relatively preserved resting spirometry, subtle decreases in diffusive capacity, increases in dead space, and changes in pulmonary mechanics translate into increased IND at rest, helping to explain the symptoms experienced by these individuals despite relatively preserved lung function (108, 129). These resting differences are exaggerated throughout exercise, with decreased exercise endurance, increased IND, and increased dyspnea in smokers-at-risk and mild COPD vs. health (108, 129). This increased dyspnea has recently been linked to ventilatory inefficiency causing premature mechanical constraint, with individuals with DLCO lower than the lower limit of normal (LLN) experiencing a higher ventilatory requirement and thus greater dyspnea and exercise intolerance than patients with DLCO > LLN despite equivalent spirometry (130). This topic is covered in greater detail in the accompanying review by (104). “Dyspnea and Exercise Limitation in Mild COPD: the value of CPET.”

## New Frontiers For Neural Drive Measurement

### Evaluating Responses to Interventions

In addition to providing insight into the mechanisms of exercise intolerance, IND measurement enables a more detailed mechanistic assessment of pharmacotherapeutic and other interventions. For example, bronchodilator-based improvements in neuromechanical coupling mirroring improvements in dyspnea during exercise challenges are documented in COPD (131, 132), while respiratory system unloading (i.e., helium unloading) independent of airway tone is similarly associated with improved indices of neuromuscular output (133, 134). Other interventions, such as supplemental O_2_ therapy or opiates, are specifically targeted at decreasing IND rather than altering respiratory mechanics (135, 136). Thus, the measurement of EMGdi in research settings can provide valuable information about IND, ultimately helping to better inform clinical approaches targeted at improving exercise performance and/or dyspnea. A possible application would include the measurement of EMGdi alongside respiratory mechanics (e.g., as outlined in the accompanying review by (54) “Non-invasive evaluation of dynamic respiratory mechanics”) to help evaluate pulmonary rehabilitation interventions targeting sarcopenia or the deconditioning of aging or chronic respiratory disease.

One application where this approach has been increasingly applied is in the evaluation of improvements in dyspnea and reductions in IND following inspiratory muscle training (IMT), proposed to occur due to improved neuromechanical coupling (137). As different IMT protocols have been assessed in diverse populations, these studies have yielded equivocal results. This includes no improvements in IND despite improvements in dyspnea and maximum inspiratory pressure when used by healthy young adults (138) or improved (decreased) IND despite maintained V_E_ and breathing pattern in COPD with baseline inspiratory muscle weakness (137). Differences in IMT study outcomes may also in part be due to the preferential recruitment of accessory muscles of inspiration during different IMT approaches and resulting breathing patterns (51, 138). Use of EMGdi measurement during IMT performed with inspiratory threshold training has shown this approach to generate better diaphragmatic recruitment and activation than IMT performed using inspiratory resistive devices in severe COPD with inspiratory muscle weakness (74, 139), while focused instruction outlining diaphragmatic breathing strategies similarly improves diaphragmatic activation during IMT in health (140). Pursed-lip breathing, a commonly employed intervention linked with improved symptoms of dyspnea and resulting in deeper and slower breathing patterns, has also been associated with reduced diaphragmatic recruitment and increased engagement of accessory muscles in advanced COPD (141). These types of targeted investigations may help optimize future rehabilitation approaches (142), and further investigation is needed to clarify those results attributable to training protocol vs. those linked directly to between-population differences.

### Applying IND Measurement in Non-CPET Settings

Emerging interest lies in the measurement of IND within novel areas of research. Two with promise are sleep and acute exacerbations of COPD. IND measurement can successfully differentiate periods of central vs. obstructive sleep apnea (143), while continuous monitoring of overnight EMGdi shows greater decreases in IND in the transition from wakefulness to non-rapid eye movement (NREM) and REM sleep in COPD vs. health, possibly holding clues to the nocturnal hypoventilation commonly observed in COPD (144). More recent work has shown the benefits of nocturnal bronchodilator therapy in improving overnight IND and respiratory mechanics (145). IND monitoring has also generated interest as a possible means of predicting recovery from acute exacerbations, with failure of acutely increased parasternal EMGdi to return to baseline conditions after hospitalization for exacerbation strongly correlated with failure to experience subjective improvements in dyspnea (Borg), lack of clinical improvement, and likelihood of readmission (146).

### Overcoming Barriers to Clinical Adoption

The integration of IND measurement into clinical settings has historically been limited by the cost of one-time use electrodes, the relative invasiveness and complexity of crural measurement approaches, challenges in standardizing measurements between visits or between individuals, and the significant technical complexities and time requirements associated with existing manual analysis approaches (39, 70). Advances in surface assessment of parasternal EMG hold significant promise for overcoming the technical barriers and patient burden associated with esophageal catheter use. This has already been successfully employed in diverse and vulnerable populations, including pediatric asthma (147), and may form the foundation of more routine adoption of IND assessment in clinical practice. The reporting of normalized values, regardless of approach, also helps to account for possible differences in signal detection between testing sessions (64).

Addressing concerns surrounding complex and time-consuming analysis approaches, significant computational advances now enable semi-automated analyses of crural EMGdi (63) as well as novel approaches to IND assessment via diaphragmatic signal entropy (148, 149), significantly improving analysis speed and consistency. Further, there is promise in the fully automated, real-time integration of IND information to inform mechanical ventilation approaches through EMGdi-based or non-invasive Neurally Adjusted Ventilatory Assist (150–152). The ongoing refinement of these approaches provides fertile ground for a more seamless integration of IND measurement into standard care. A final requirement for the translation of IND from research to clinical laboratories is the establishment of normative resting and exercise values of EMGdi in both sexes across age groups. Until such values are available, the use of age- and sex-matched comparator populations is essential in the investigation of disease.

## Conclusions

As far back as 1929, the *Lancet* submitted a “plea for a careful clinical study of the diaphragm in chest disease” (153). In the century that has followed, significant progress has been made in elucidating not only the structure, but increasingly the function, of our primary pump muscle. The foundation that has been laid surrounding the utility of EMGdi as a marker of IND and its associated sequelae of dyspnea and exercise limitation is now well-positioned for translation into clinical practice. The ability of IND to reflect alterations in ventilatory load and capacity holds significant promise for its possible use as a global marker of disease severity and ventilatory dysfunction, as well as a useful target for monitoring the success of therapeutic interventions.

## Author Contributions

ND contributed to planning and drafting of the submission. ND, EW, and DL all contributed to editing of the article and approved the submitted version.

## Conflict of Interest

The authors declare that the research was conducted in the absence of any commercial or financial relationships that could be construed as a potential conflict of interest.
